# A novel assay to detect calreticulin mutations in myeloproliferative neoplasms

**DOI:** 10.18632/oncotarget.14113

**Published:** 2016-12-23

**Authors:** Valentina Rosso, Jessica Petiti, Enrico Bracco, Roberto Pedrola, Francesca Carnuccio, Elisabetta Signorino, Sonia Carturan, Chiara Calabrese, Giada Bot-Sartor, Michela Ronconi, Anna Serra, Giuseppe Saglio, Francesco Frassoni, Daniela Cilloni

**Affiliations:** ^1^ Department of Clinical and Biological Sciences, University of Turin, Turin, Italy; ^2^ Department of Oncology, University of Turin, Turin, Italy; ^3^ Department of Pediatric Hemato-Oncology and Stem Cell, Cellular Therapy Laboratory, Institute G. Gaslini, Genova, Italy

**Keywords:** CALR, PNA, MPN, PCR clamping, diagnostic assay

## Abstract

The myeloproliferative neoplasms are chronic myeloid cancers divided in Philadelphia positive (Ph+), chronic myeloid leukemia, or negative: polycythemia vera (PV) essential thrombocythemia (ET), and primary myelofibrosis (PMF). Most Ph negative cases have an activating JAK2 or MPL mutation. Recently, somatic mutations in the calreticulin gene (*CALR*) were detected in 56–88% of *JAK2/MPL*-negative patients affected by ET or PMF. The most frequent mutations in CARL gene are type-1 and 2. Currently, *CALR* mutations are evaluated by sanger sequencing. The evaluation of *CARL* mutations increases the diagnostic accuracy in patients without other molecular markers and could represent a new therapeutic target for molecular drugs.

We developed a novel detection assay in order to identify type-1 and 2 *CALR* mutations by PNA directed PCR clamping. Seventy-five patients affected by myeloproliferative neoplasms and seven controls were examined by direct DNA sequencing and by PNA directed PCR clamping. The assay resulted to be more sensitive, specific and cheaper than sanger sequencing and it could be applied even in laboratory not equipped for more sophisticated analysis. Interestingly, we report here a case carrying both type 1 and type2 mutations in *CALR* gene.

## INTRODUCTION

The myeloproliferative neoplasms (MPN) are chronic myeloid cancers characterized by the overproduction of mature blood cells and risk of transformation into acute myeloid leukaemia (AML) [1, 2]. In addition to chronic myeloid leukaemia (CML) characterized by the *BCR-ABL* fusion gene, the three most common myeloproliferative neoplasms are essential thrombocythemia (ET), polycythemia vera (PV), and primary myelofibrosis (PMF). In 2005 the JAK2 V617F mutation has been identified in patients with PV, ET and PMF [3, 4]. Mutations of *JAK2* exon 12 [5, 6] and *MPL* exon 10 [7] were subsequently detected in a subsets of patients. Recently, several studies identified additional mutations present in smaller fraction of patients [8–12]. In 2013, somatic mutations in the *CALR* gene, encoding calreticulin, were detected in most patients with ET or PMF without mutations in *JAK2* and *MPL* genes [13, 14].

Calreticulin is a protein with different functions. In the endoplasmic reticulum the protein modulates calcium homeostasis and regulates the folding of glycoproteins. [15] Calreticulin is also localized in other cellular compartments where it regulates many biological functions including apoptosis and proliferation. [16]. All mutant *CALR* proteins discovered until now share a novel amino acid sequence at the C-terminal. The non-mutant *CALR* C-terminal is mainly negatively charged, whereas the mutant *CALR* C-terminal is largely positively charged [17]. The mutant CALR protein lacks the endoplasmic reticulum retention signal (KDEL) and therefore it may have an altered subcellular localization. The role of *CALR* in the pathogenesis of MPN is largely unknown. Klampfl and colleagues and Nangalia and colleagues [13, 14] elegantly demonstrated that *CALR* mutations are acquired early in the major clone. Several studies are trying to shed light on how mutant CALR can sustain the uncontrolled proliferation. It was demonstrated that mutant CALR activates JAK/STAT signaling thus generating a proliferative signaling [13, 14]. Recently, it was shown that the forced expression of mutant *CALR* in hematopoietic cell lines resulted in MPL-dependent activation of MAPK signaling. In line whit this finding, patients with *CALR* mutations showed increased MAPK activity in blood cells and in CD34+ cells leading to enhance megakaryopoiesis and pro-platelet production. [18] Recently it was also shown the IL3 independent growth of *CALR* mutant cells [17].

*CALR* mutations have been reported as mutually exclusive with *JAK2* and *MPL* mutations and are present in a percentage of patients ranging from 56 to 88% of *JAK2/MPL*-negative cases [13, 14]. A recent paper reported a patient who carried simultaneously both mutations, *JAK2* V617F and *CALR* exon 9 [19]. Many different CALR mutations have been described in exon 9 but the most frequent is type-1 [L367fs*46; deletion of 52 base pairs (bp)] and type-2 [K385fs*47; insertion of 5 bp] [13, 14]. Currently, the method commonly exploited for the detection of *CARL* mutations is sanger sequencing.

Peptide nucleic acid (PNA) is able to hybridize very specifically to DNA. It is recognized that the binding PNA/DNA is more stable than DNA/DNA or DNA/RNA duplexes [20, 21], but PNA sequences cannot be extended by polymerase [22]. Consequently, PNA/DNA duplex suppresses DNA amplification. This is the principle that allowed to design PNA directed PCR clamping to discriminate wild type from mutated sequences [22–24].

The goal of the study was to make the diagnosis of *CALR* mutations more rapid, easy and accessible.

Based on this premise, we developed a novel and sensitive assay based on PNA directed PCR clamping for the detection of *CALR* mutations (type-1 and type-2) in MPN patients.

## RESULTS

**Figure 1:**
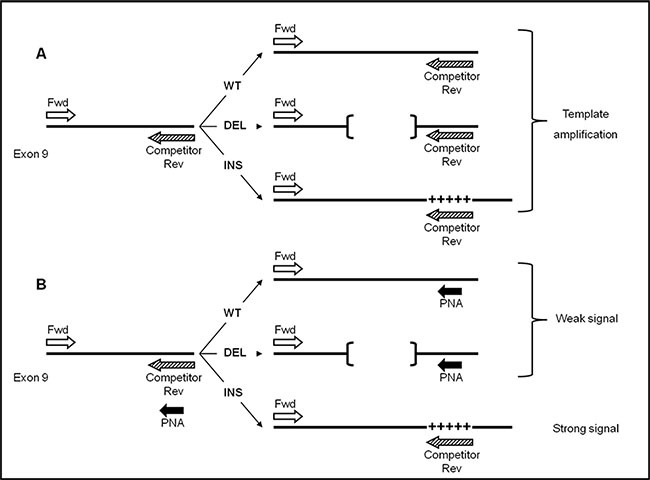
Step II: PNA clamping experimental design Template amplification of *CALR* wild-type (WT), type-1 (DEL) and type-2 (INS) mutations (**A**) in absence of PNA probe (**B**) and with PNA probe. Perfect PNA/DNA hybridization occurs when *CALR* template sequence is wild-type (WT) or type-1 mutation (DEL, indicated by [¨¨]), thus leading to a significant reduction of PCR amplification. By contrast, in presence of type-2 mutation (INS, indicated by +++++), PNA/DNA duplex is highly destabilized, on behalf of primer Competitor Rev, allowing strong template amplification.

As show in Figure 1, the method described here forecasts that both PNA probe and PCR primer competitor target sites overlap, thus leading to a direct competition towards complementary DNA. In case *CALR* sequence is wild type, a perfect matching occurs between PNA (designed on wild sequence) and DNA. As a consequence, the PNA-template hybridization is favoured and DNA amplification is strongly suppressed. This occurs in patients with *CALR* wild-type (WT) or *CALR* type-1 mutation (DEL), producing weak bands at different lengths.

**Figure 2:**
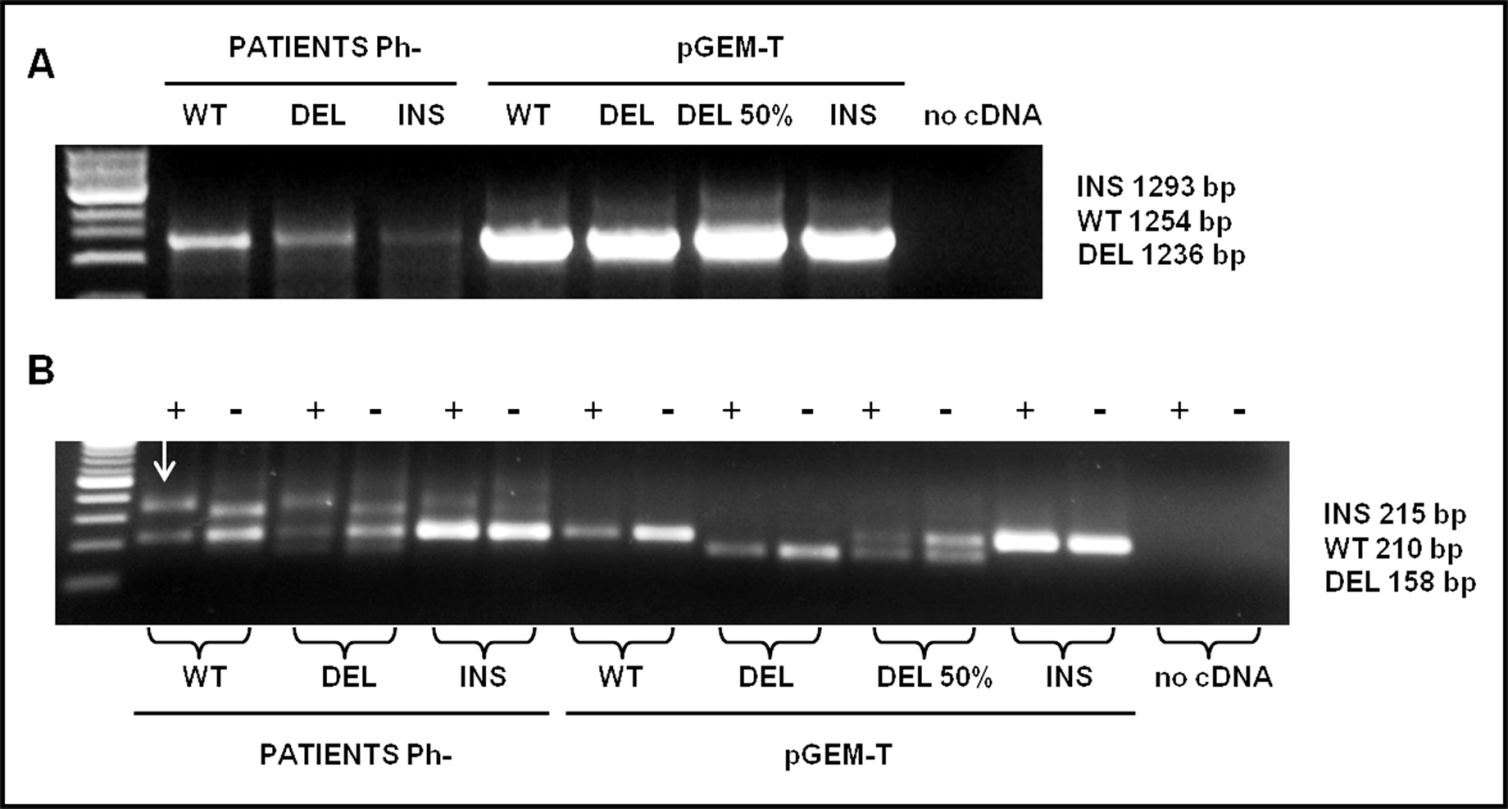
Detection analysis by PNA directed PCR clamping of CALR type-1 (DEL) and type-2 (INS) mutations (**A**) Step I: cDNA amplification of patients with *CALR* wild-type (WT), type-1 (DEL) and type-2 (INS) mutations. pGEM-T-*CALR* wild-type (WT), pGEM-T-*CALR* type-1 mut (DEL), pGEM-T-*CALR* type-1 mut 50% vs. wild-type (DEL 50%) and pGEM-*CALR* type-2 mut (INS) were used as PCR positive control. 5 μL of each amplifier were loaded on 1% Agarose-TBE 1x gel with 5 μg/mL ethidium bromide (EtBr) and run at 120 V for 30 minutes. (**B**) Step II: PCR amplification of a small area of *CALR* gene, containing type-1 and type-2 mutations, was carried-out in absence (−) or in presence (+) of PNA probe. The plasmids amplified in the step I were used, in a dilution of 1:100, in the step II in order to interpret the results, 10 μL of each amplifier were loaded on 2% Agarose-TBE 1x gel with 5μg/mL EtBr and run at 100 V for 30 minutes and than at 65 V for 15 minutes. The arrow indicates the non-specific band present in the amplified of patients.

The lengths of the bands allow to distinguish between *CALR WT* and *DEL* (Figure 2B). Conversely, a non-perfect matching ( as in case of a mutation) destabilizes the PNA hybridization thus favouring the hybridization between template and primer competitor. In this case an amplification product is evident. (as in the case of patients with *CALR* type-2 mutation (INS)) (Figure 2B).

There are two PCR steps. Through the first step, the selected *CALR* sequence is amplified (Figure 2A). The second step is carried-out in duplicate, in the presence (+) or in the absence (-) of the PNA probe. The final result should be interpreted by reading the double loading for each individual patient (Figure 2B).

**Figure 3:**
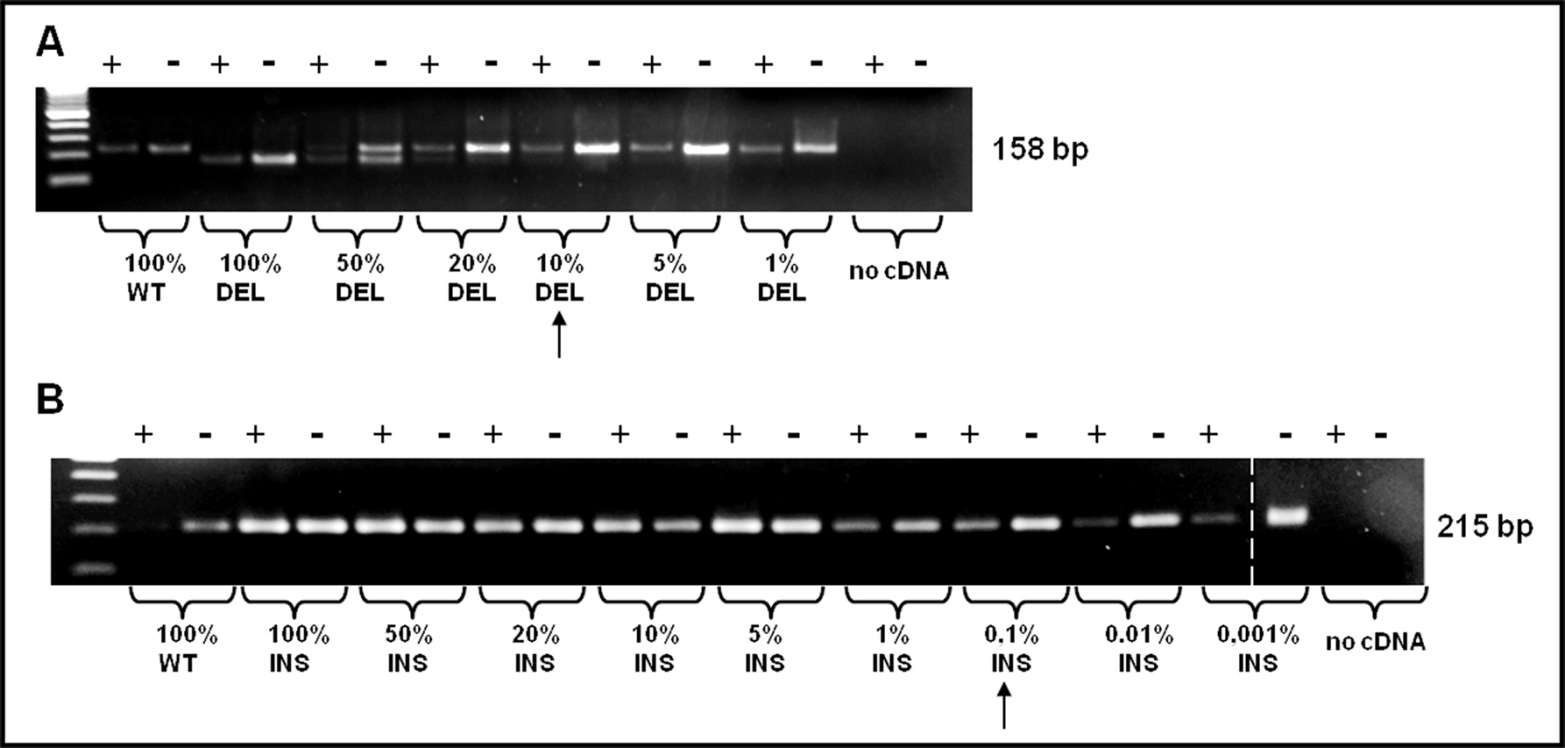
PNA directed PCR clamping sensitivity was assessed mixing, at different ratio, mutated (type-1 and type-2) and WT template The arrows indicate the sensitivity of the method. (**A**) Dilutions were as follow: 100, 50, 20, 10, 5, 1% *CALR* type-1 mutation vs. WT template and (**B**) 100, 50, 20, 10, 5, 1, 0,1, 0,01, 0,001% *CARL* type-2 mutation vs. WT template. The dashed vertical bar separates different parts of the same gel.

As shown in Figure 2B a non-specific band appears in patients approximately 100 bp higher than the WT band; however, it will not compromise the interpretation of the result. As shown in Figure 3, sensitivity was assessed mixing, at different ratio, mutated (type-1 and type-2) and WT template. Dilutions were as follow: 100, 50, 20, 10, and 5 % type-1 deletion (52 bp) mutated versus WT template and 100, 50, 20, 10, 5, 1, 0.1, 0.01, 0.001% type-2 insertion (5 bp) mutated versus WT template. The method displays a quite high sensitivity, allowing to detect an amount of mutated template as low as 10% for *CALR* type-1 and 0,1% for *CALR* type-2 mutations, which cannot be identified by sanger sequencing. Our diagnostic test shows a sensitivity of 100% (CI 79.41% to 100.00%) and a specificity of 98,5% (CI 91.96% to 99.96%) with an AUC corresponding to 0.99.

**Figure 4:**
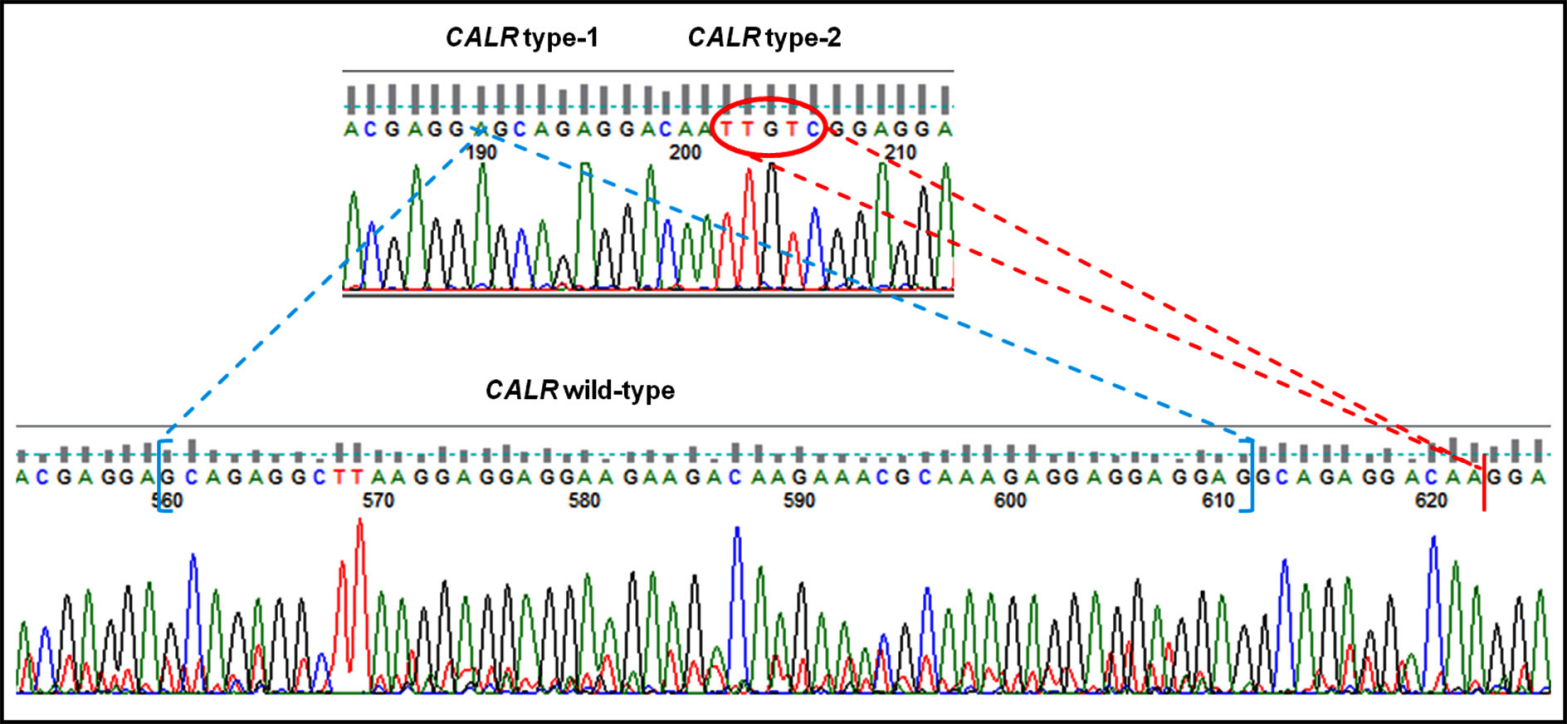
Sequencing chromatogram of patient with both CARL type-1 and 2 mutations compared with WT sequence *CALR* type-1 mutation is indicated in blue, while *CALR* type-2 is highlighted in red.

Seventy-five patients affected by MPN and seven controls were examined in double blind either by direct DNA sequencing or by PNA directed PCR clamping, displaying identical readout except for two samples: mutated (type-1 mutation) in PNA directed PCR clamping, but wild-type by sanger sequencing. For these patients, the experiment was repeated and the shorter bands, resulting by step II of PNA directed PCR clamping, were purified and cloned in pGEM-T Easy Vector as previously described. Then, they were sequenced by sanger method using T7 and SP6 primers (respectively #Q5021 e #Q5011, Promega, Milan, Italy): one of these resulted wild-type and represents the only false positive sample tested; unexpectedly, the second one was found to have both the *CALR* type-1 and 2 mutations (Figure 4).

## DISCUSSION

The majority of Ph- myeloproliferative neoplasms shows mutations in *JAK2* or *MPL* genes. Recently, additional mutations were identified in ET and PMF wild-type for *JAK2* and *MPL*. We developed a novel molecular assay, based on PNA direct PCR clamping, for a rapid diagnosis of *CALR* type-1 and 2 mutations in patients affected by MPN. The assay shows high efficiency, sensitivity and specificity. Our method appears to be faster, cheaper and easily interpretable compared with DNA sanger sequencing, the method most commonly used for the detections of these mutations. In clinical practice, the evaluation of *CARL* mutations increases the diagnostic accuracy of ET and PMF and allows a more accurate diagnosis in patients without other molecular markers. Greater accuracy of diagnosis can result in a more precise prediction of prognosis and in a more effective therapeutic choice [25, 26]. Furthermore, the mutated protein CALR could represent a new therapeutic target for molecular drugs [25]. Contrary to *JAK2* V617F mutation, a correlation between *CALR* mutant allele quantification and clinical presentation has not yet been demonstrated [27]. Despite the sanger sequencing is the most commonly method used to diagnose these mutations, many laboratories in the world are not equipped with such a facility or cannot effort the cost of this procedure. In conclusion, considering the diagnostic relevance of the *CALR* mutations, the PNA direct PCR clamping assay might represent a good alternative method for the detection of *CALR* type-1 and type-2 mutations, especially considering the costs and the lower sensitivity of the DNA sequencing method. Furthermore, because this assay is faster, more sensitive, and less expensive than sanger sequencing, it might be applied also in laboratory not equipped for sophisticated analysis, reducing costs and time for an advanced molecular diagnosis.

## MATERIALS AND METHODS

### Cells sources and RNA extraction

After informed consent, peripheral blood were collected at diagnosis from 82 patients: 75 patients affected by myeloproliferative disease and 7 controls (5 healthy donors and 2 patients affected by myelodysplastic syndromes). The study was approved by the ethic committee on December 16^th^ 2015 (number of approval 212/2015). Table 1 summarizes clinical and molecular characteristics of patients.

**Table 1:** Clinical and molecular characteristics of patients

Disease	n° of pts	% of *JAK2* mut pts	% of *CALR* mut pts
PV	25	28	8
ET	38	13	40
PMF	12	17	25
Controls	7	0	0

PV: polycythemia vera; ET: essential thrombocythemia; PMF: primary myelofibrosis

Total RNA was extracted from buffy-coat fraction using TRIzol Reagent (Ambion, Thermo Fisher Scientific, Massachusetts, USA), following the manufacturer's instructions, and 1 μg was reverse transcribed using random hexamers as primers in a final volume of 25 μL.

### Cloning PCR controls with pGEM^®^-T easy vector

*CALR WT, CALR DEL* and *CALR INS* were amplified and purified by QIAgenquick Gel Extraction Kit (Qiagen, Hildem, Germany) and cloned in pGEM-T Easy Vector (Promega, Milan, Italy). All reactions were performed following the manufacturer's instructions. Plasmids were used as positive control in PCR steps. Primers sequences are listed in Table 2.

**Table 2:** Primers sequences used for cloning and for sanger sequencing

**Cloning**	***CALR* Fwd**	5'–ATGCTGCTATCCGTGCCGCTGCT–3'
	***CALR* Rev WT**	5'–CTACAGCTCGTCCTTGGCCTGGC–3'
	***CALR* Rev Mut**	5'–TCAGGCCTCAGTCCAGCCCTGGA–3'
**Sequencing**	***CALR* Fwd**	5'–ACAACTTCCTCATCACCAACG–3'
	***CALR* Rev**	5'–GGCCTCAGTCCAGCCCTG–3'

### Capillary sanger sequence method for the detection of *CALR*

Detection of *CALR* mutations was performed by capillary sanger sequence method. DNA was extracted by Maxwell 16 (Promega, Milan, Italy) following the manufacturer's protocol. *CALR* was amplified and analyzed by sequencing with BigDye terminator v3.1 (Applied Biosystem, Foster City, California) and capillary electrophoresis on ABI PRISM 3130XL (Applied Biosystem, Foster City, California). Primers sequences are listed in Table 2. The sensitivity was previously estimated by serial dilutions experiments to be 20%.

### PNA directed PCR clamping

Step I: 3 μL of cDNA were used to amplify the *CALR* mRNA sequence (acc. # NM_004343) and ~10 ng of pGEM-T-*CALR* wild-type (WT), pGEM-T-*CALR* type-1 mut (DEL), pGEM-T-*CALR* type-1 mut 50% vs. wild-type (DEL 50%) and pGEM-*CALR* type-2 mut (INS) were used as PCR positive control.

Primers sequences were as follow (step I):

Fwd: 5'–ATGCTGCTATCCGTGCCGCTGCT–3'

Rev: 5'–TCAGGCCTCAGTCCAGCCCTGGA–3'

Reaction volume was 50 μL and the final concentrations of the reagents were the following: MgCl_2_ [2, 5 μM], 10X PCR Buffer [1X], dNTPs [0,4 μM], AmpliTaq 2U, Primers [0, 6 μM], DMSO 10% , H_2_O MilliQ.

Step II: 1μL of step I PCR product was used to amplify a small area of *CALR* gene, containing type-1 and type-2 mutations. PCR amplification was carried-out in absence (−) or in presence (+) of PNA probe, at a concentration 6 × greater than primer Competitor Rev. The amplification performed without (−) PNA probe represents an internal positive control displaying the PCR efficiency.

Primers and PNA probe sequences were as follow (step II):

Fwd: 5'–CCTCTGGCAGGTCAAGTCTG–3'

Rev Competitor: 5'– ATCCTCCGACAATTGTCCT CTG–3'

PNA probe: 5'–OO–CATCCTCCTTGTCCTC–3'

Reaction volume was 50 μL and he final concentrations of the reagents were the following:

MgCl2 [1, 25 μM], 10X PCR Buffer [1X], dNTPs [0, 2 μM], AmpliTaq 1U, Primers [0, 2 μM], PNA probe [1, 2 μM].

PNA probe, designed on the human *CALR* cDNA (acc. # NM_004343), encompasses a very short sequence in exon 9, common to both the wild-type and type-1 mutation sequence. PNA-PCR clamping conditions for *CALR* mutations detection were as follow: step I PCR: 94°C 3 min, (94°C 30 s, 65°C 40 s, 72°C 1 min 30 s) for 35 cycles, 72°C 5 min; step II PCR: 94°C 3 min, (94°C 30 s, 55°C 27 s, 72°C 45 s) for 30 cycles.

Diagnostic test valuation with a confidence interval (CI) of 95% and the area under the curve (AUC) were calculated with MedCalc software (MedCalc, Osten, Belgium).

The second step of this assay has been patented by the authors and by the University of Turin (patent pending, number 102016000042586).

VR is a fellow of Compagnia di San Paolo.

JP is a fellow of Gigi Ghirotti Foundation.
